# Mitotic redistribution of the mitochondrial network by Miro and Cenp-F

**DOI:** 10.1038/ncomms9015

**Published:** 2015-08-11

**Authors:** Gil Kanfer, Thibault Courthéoux, Martin Peterka, Sonja Meier, Martin Soste, Andre Melnik, Katarina Reis, Pontus Aspenström, Matthias Peter, Paola Picotti, Benoît Kornmann

**Affiliations:** 1 Institute of Biochemistry, ETH Zürich, 8093 Zürich, Switzerland; 2 Department of Microbiology, Tumor and Cell Biology, Karolinska Institutet, SE-171 77, Stockholm, Sweden

## Abstract

Although chromosome partitioning during mitosis is well studied, the molecular mechanisms that allow proper segregation of cytoplasmic organelles in human cells are poorly understood. Here we show that mitochondria interact with growing microtubule tips and are transported towards the daughter cell periphery at the end of mitosis. This phenomenon is promoted by the direct and cell cycle-dependent interaction of the mitochondrial protein Miro and the cytoskeletal-associated protein Cenp-F. Cenp-F is recruited to mitochondria by Miro at the time of cytokinesis and associates with microtubule growing tips. Cells devoid of Cenp-F or Miro show decreased spreading of the mitochondrial network as well as cytokinesis-specific defects in mitochondrial transport towards the cell periphery. Thus, Miro and Cenp-F promote anterograde mitochondrial movement and proper mitochondrial distribution in daughter cells.

## Abstract (web summary)

During mitosis, mitochondria partition into daughter cells through microtubule-based transport. Here the authors show that the mitochondrial protein Miro and the cytoskeletal-associated protein Cenp-F interact in a cell-cycle dependent manner to promote microtubule-directed movement of mitochondria.

## Graphical abstract

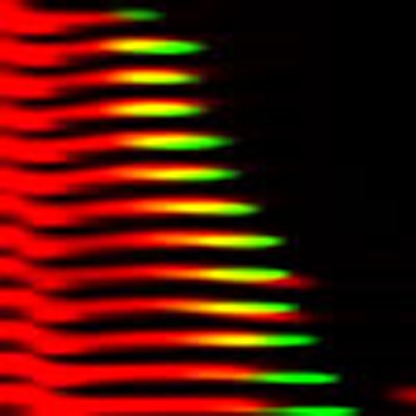

Mitosis requires profound cytoskeletal reorganization to ensure correct chromosome partitioning. This causes marked changes in cell shape and in the distribution of the cytoplasmic organelles. For example, during mitosis, mitochondria fragment^1^,^2^ and congress towards the dividing cell centre before partitioning into daughter cells through microtubule-based transport. This partitioning likely involves linking mitochondria to molecular motors.

To recruit molecular motors, mitochondria have specialized adaptors on their outer membrane. The best-characterized adaptor is the transmembrane calcium-binding GTPase Miro, which plays an essential role in mitochondrial trafficking along axons^3^,^4^,^5^,^6^. Yet, how mitochondria are distributed to daughter cells after mitosis remains unknown.

## Results

### Miro GTPases interact with the centromeric protein F

To identify Miro interactors, we established stable HEK293 cell lines that expressed doxycycline-inducible 3 × Flag-tagged versions of the two Miro paralogues encoded in the human genome—Miro1 and Miro2—and used them for immunoprecipitation (Supplementary Fig. 1A,B) coupled to mass spectrometry. This analysis identified Miro2 in FLAG-Miro1 pull-downs and vice versa, indicating that Miro1 and Miro2 are part of the same complex and underscoring the validity of our approach (Supplementary Data Set 1). This analysis also identified a large number of unrelated proteins, indicating possible unspecific interactions. We therefore devised a stable-isotope amino-acid labelling in culture (SILAC) strategy^7^. We mixed cells expressing Flag-tagged Miro1 with ^15^N-^13^C-Arg/^15^N-^13^C-Lys-labelled non-transgenic cells, prior to lysis and immunoprecipitation (Supplementary Fig. 1C). In such conditions, illegitimate proteins that bind Miro1 or the immunoprecipitation matrix consequent to cell lysis should derive from both the heavy-labelled and the non-labelled proteins, while *bona fide* interactors should be enriched for non-labelled proteins. Using this approach, we detected only three proteins (Supplementary Fig. 1D) that showed non-labelled peptides enrichment: Miro1, Miro2 and centromeric protein F (Cenp-F).

Cenp-F came as a surprise. It is a large (367 kDa) coiled-coil protein that accumulates during the G2 cell cycle phase, culminating during mitosis^8^. Cenp-F functions at the kinetochore and participates in nuclear envelope disassembly. Cenp-F interacts with several cytoskeletal components, such as Dynein motor complexes and microtubules themselves^9^,^10^,^11^,^12^,^13^.

### Cenp-F localizes at mitochondria–microtubule interfaces

**Figure 1:**
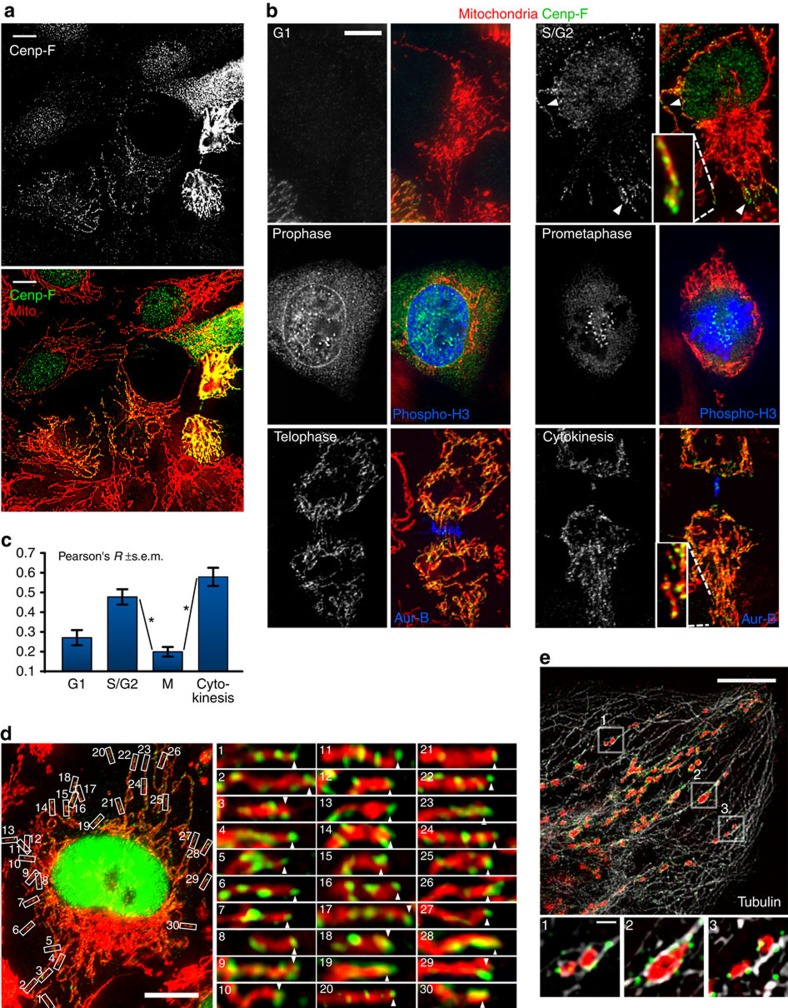
Cenp-F localizes to mitochondria in a cell cycle-dependent manner. In all panels, mitochondria are shown in red and Cenp-F in green. (**a**) Cenp-F staining is heterogeneous in a field of unsynchronized cells. Immunofluorescence using an α-Cenp-F antibody (green in lower panel) of KERMIT cells expressing a mitochondrial marker (mtBFP, red in lower panel). Scale bar, 5 μm. (**b**) As in **a**. Where indicated, cells were labelled with cell-cycle markers: α-Phosphorylated histone H3 (phospho-H3, blue) α-Aurora-B (Aur-B, blue). Interphase cells were classified as G1 and S/G2 based on Cenp-F levels. Scale bar, 5 μm. (**c**) Quantification of Cenp-F–mitochondria colocalization in different cell cycle phases. M represents mitosis, from prometaphase to anaphase, *P value <10^−6^ from a Mann–Whitney–Wilcoxon *U*-test. For quantifications, 2–4 regions were selected per cell. Number of selected regions: G1: 14; S/G2: 39, M: 48, cytokinesis: 17. The experiment was repeated at least three times. (**d**) Cenp-F localizes to mitochondrial tips. As in **a**. Scale bar, 10 μm. The right panels are higher magnifications of the boxed area. (**e**) Cenp-F colocalizes with mitochondria and microtubules. Structured-illumination microscopy of U2OS cells stained with mitotracker-red (red), Cenp-F (green) and tubulin-α (white). Scale bars, 5 μm (top panel), 500 nm (bottom).

To assess whether Cenp-F localized to the mitochondria, we generated a U2OS-derived stable cell line co-expressing a mitochondrial and an ER marker (mtBFP and sec61α-GFP, respectively), which we called KERMIT (for Kinesis of ER and MITochondria). Cenp-F displayed highly variable immunofluorescence staining from cell to cell (Fig. 1a), likely due to the fact that cells were at different cell cycle phases. We imaged Cenp-F together with cell cycle markers, including cyclin A that accumulates in G2 and is degraded in mitosis, phosphorylated histone H3 (Phospho-H3) that is a marker of mitotic chromatin and Aurora-B (Aur-B) that localizes to the midbody during cytokinesis^14^. Cenp-F showed expected localization patterns; Cenp-F was undetectable in G1 and was found in the nucleus, the nuclear envelope, the kinetochores and the midbody in G2, early prophase, (pro)metaphase and cytokinesis, respectively (Fig. 1b; Supplementary Fig. 2A,B). In addition, in S/G2, a fraction of Cenp-F was found on mitochondria (Fig. 1b, top right). Strikingly, at the end of mitosis, Cenp-F was strongly recruited to mitochondria (Fig. 1b, bottom). The profound difference in recruitment between early and late mitosis (Fig. 1c) was not merely due to differences in Cenp-F levels (Supplementary Fig. 2C), and must therefore indicate a regulated process.

Interestingly, during S/G2, Cenp-F was enriched at the distal tip of mitochondria projecting away from the cell centre (Fig. 1d; Supplementary Fig. 2D). This, together with the fact that Cenp-F is a microtubule-binding protein, suggested that Cenp-F mediated mitochondria–cytoskeleton interaction. We used structured-illumination super-resolution microscopy (3D-SIM) to assess the relationship between mitochondria, Cenp-F and microtubules. Microtubules are best conserved by methanol fixation. This fixation protocol, however, does not preserve the fluorescence of the mitochondrial marker in KERMIT cells (our observations). To circumvent this problem, we used U2OS cells stained with mitotracker Red. We imaged S/G2 cells, because (1) they were Cenp-F-positive and (2) the microtubule network is much better imaged in flat interphase cells, compared with round mitotic cells. Virtually all mitochondrial Cenp-F puncta colocalized with microtubules (Fig. 1e), indicating that Cenp-F is at the mitochondria–microtubules interface.

### Miro directly recruits Cenp-F to mitochondria

**Figure 2:**
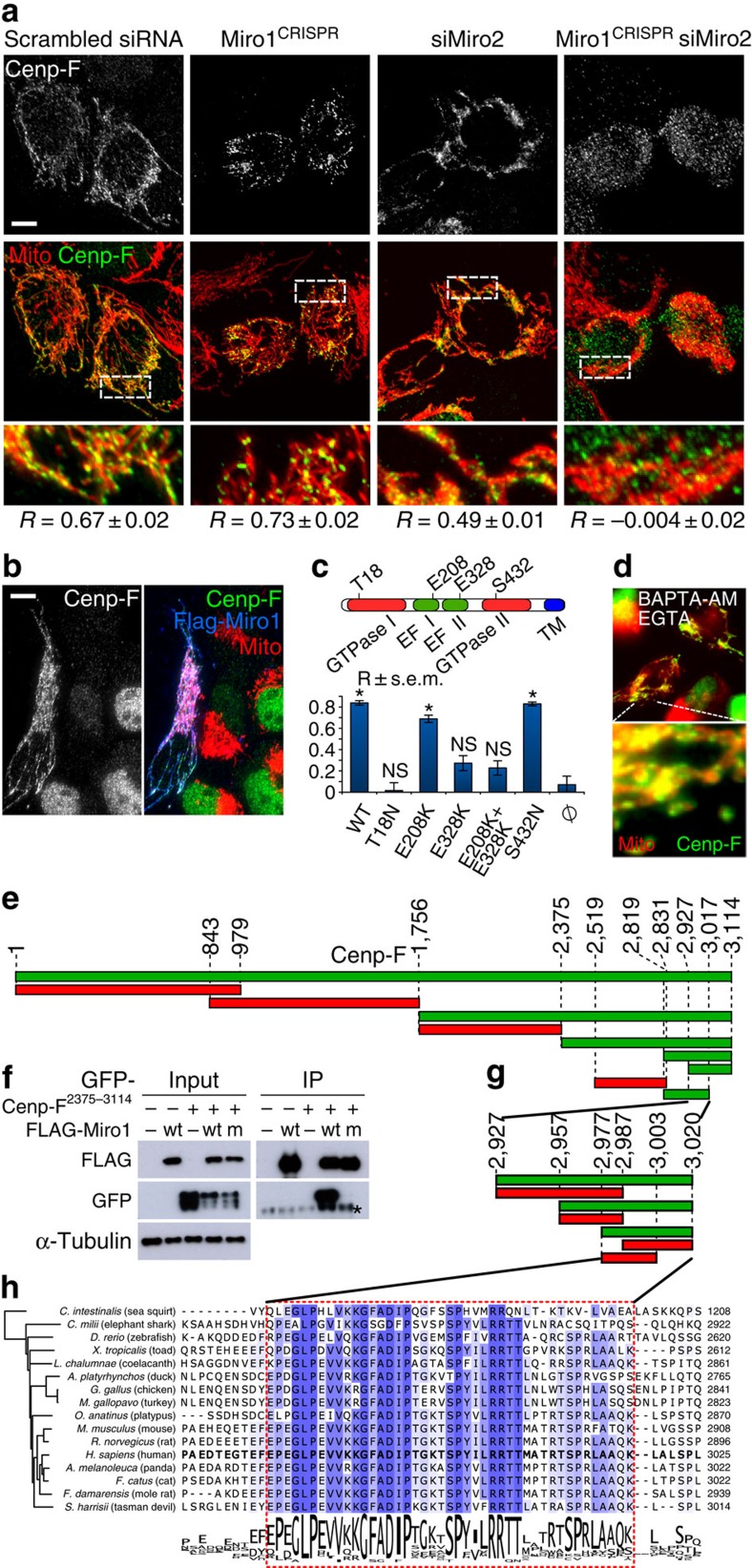
Miro recruits Cenp-F to mitochondria. (**a**) Miro is necessary for Cenp-F mitochondrial recruitment. Localization of Cenp-F (green) and mitochondria (red) in the presence and absence of Miro1, Miro2 or both. Top: Cenp-F signal only. Middle: overlay. Bottom: magnification of the boxed areas. R: Pearson coefficient colocalization analysis±s.e.m. Scale bar, 10 μm. Two to four regions were selected per cell. Number of selected regions: Miro1^CRISPR^: 98; siMiro2: 36; Miro1^CRISPR^-siMiro2: 34. The experiment was repeated at least four times. (**b**) Miro is sufficient for Cenp-F recruitment. Immunofluorescence of Miro-less cells overexpressing FLAG-Miro1 (blue). Green: Cenp-F, red: mitochondria. Scale bar, 10 μm. (**c**) Cenp-F recruitment to mitochondria by Miro1 mutants. Miro-less cells were transfected as in **b** with plasmids encoding the indicated mutants of Miro1. Recruitment was scored using the Pearson’s *R* coefficient. Ø: untransfected cells. *P value <10^−7^ (compared with untransfected cells) from a Mann–Whitney–Wilcoxon *U*-test. NS, non-significant. Quantifications as in **a**. Number of selected regions: WT: 29; T18N: 17; E208K: 33; E328K: 28; E208K+E328K: 28; S432N: 31; ϕ: 27. The experiment was repeated at least four times. (**d**) Cytosolic calcium withdrawal does not prevent Cenp-F mitochondrial recruitment. Cells treated with EGTA and BAPTA-AM to chelate extra- and intracellular calcium were imaged as in **a**. (**e**) Mapping of the Miro-interaction domain on Cenp-F. KERMIT cells were cotransfected with FLAG-Miro and GFP fusion of indicated Cenp-F fragments. Green fragments are recruited to mitochondria. Red ones are not. (**f**) Co-immunoprecipitation of transiently transfected Cenp-F fragment (amino acids 2375–3144) and wild-type (wt) and calcium-binding mutant (m) Miro1. * IgG shedding from the beads. (**g**) Yeast two-hybrid mapping of the Miro-interaction domain on Cenp-F. Green fragments are positive for interaction. Red ones are not. (**h**) Alignment of the Miro-binding domain of Cenp-F in chordates. The phylogenetic tree is generated by neighbour joining using percentage identity.

**Figure 3:**
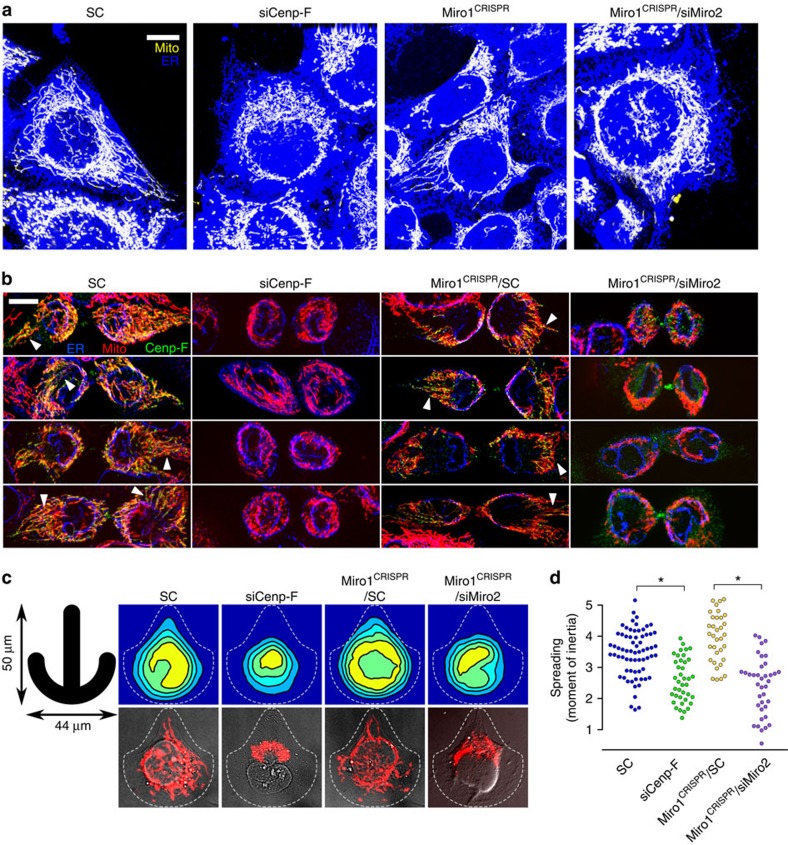
Miro and Cenp-F influence mitochondrial distribution. (**a**) KERMIT cells of the indicated genotype with the indicated treatment, imaged both for mitochondria (mtBFP, yellow) and the ER (sec-61α-GFP, blue), which indicates cell boundaries. SC, scrambled siRNA. Scale bar, 10 μm. (**b**) Immunofluorescence of cytokinetic KERMIT cells with α-Cenp-F antibody. Arrowheads indicate bundles of parallel mitochondria. Scale bar, 10 μm. (**c**) Live KERMIT cells of the indicated genotype with the indicated treatment were seeded on crossbow-shaped micropatterns (left). Top: probability density maps of the position of the mitochondrial network. Bottom: example cells. The dashed line shows typical cell boundaries. (**d**) Measurement of the moment of inertia of the mitochondrial network of the cells in **c**. *P<10^−5^ from a Mann–Whitney–Wilcoxon *U*-test. The experiment has been repeated at least three times.

We wondered whether Miro was responsible for recruiting Cenp-F to mitochondria. To achieve complete loss-of-function of both Miro1 and Miro2, we mutated Miro1 using the CRISPR/Cas9 technology in KERMIT cells^15^. We then knocked down Miro2 using specific small interfering RNAs (siRNAs). We generated four Miro1 CRISPR clones, bearing different homozygous mutations in exon 7 and 8. Two were used for subsequent studies (Supplementary Fig. 3A). Miro2 knockdown in Miro1-deficient cells (hereafter, Miro-less cells) led to a complete disappearance of Cenp-F from the mitochondria, even in cytokinetic cells (Figs 2a and 3b; ; Supplementary Fig. 3B), while the localization to the nucleus, nuclear envelope, kinetochores and midbody was unaffected (Supplementary Fig. 3B). Thus, Miro is necessary for Cenp-F recruitment to mitochondria. Conversely, overexpressing FLAG-Miro1 (Fig. 2b) or FLAG-Miro2 (Supplementary Fig. 3C) in KERMIT cells increased mitochondrial recruitment of Cenp-F, showing that Miro is also sufficient for Cenp-F mitochondrial recruitment.

Miro bears two calcium-binding EF hands that regulate its association to molecular motors^4^, and two GTPase domains of unclear function^16^. To assess whether any of these domains were involved in Cenp-F recruitment, we transfected Miro-less cells with FLAG-Miro1 bearing mutations predicted to abolish GTPase activity and calcium binding^16^. Mutating the first GTPase domain (T18N) abrogated Cenp-F recruitment, while mutating the second domain (S462N) had no effect. Mutating the EF hands (E208K and E328K) strongly reduced Cenp-F recruitment (Fig. 2c). However, lowering cytosolic calcium level with EGTA and BAPTA-AM did not produce the same effect (Fig. 2d; Supplementary Fig. 3E), indicating that the predicted Miro calcium-binding mutants likely did not mimic calcium-free Miro, and suffered from additional defects. This finding reveals an important caveat in the usage of such mutants to mimic calcium-free Miro^4^,^5^,^16^. To define which part(s) of Cenp-F interacts with Miro, we fused all or parts of Cenp-F to GFP. Although GFP-Cenp-F^17^ did not recapitulate all the features of endogenous Cenp-F localization, it was robustly recruited to mitochondria by FLAG-Miro1 co-overexpression (Supplementary Fig. 3F), allowing us to narrow down the interaction domain to a C-terminal fragment (Fig. 2e). For Cenp-F fragments showing mitochondrial recruitment in cells, we could reconstitute the interaction in pull-down (Fig. 2f) and yeast two-hybrid assays (Supplementary Fig. 3G). This latter assay showed that Cenp-F directly interacts with both Miro1 and Miro2, and allowed us to narrow the binding domain (Fig. 2g) to a highly conserved 40-amino-acid peptide (Fig. 2h).

### Miro and Cenp-F are important for mitochondrial distribution

We hypothesized that Miro-dependent mitochondrial Cenp-F recruitment could serve in mitochondrial transport and distribution in the cell. This idea is consistent with a recent study reporting that mitochondria fail to spread to the cell periphery in Miro1 knockout mouse embryonic fibroblasts^6^. We observed that indeed, mitochondria extended less towards the periphery in cells lacking both Miro paralogues (Fig. 3a, right). In our case, no phenotype was observed in cells lacking Miro1 only, indicating that the degree of redundancy of Miro1 and Miro2 may vary with cell type. Interestingly, the spreading defect observed in Miro-less cells was phenocopied in Cenp-F-silenced cells (Fig. 3a; Supplementary Fig. 3D).

Yet, because Cenp-F is strongly recruited to mitochondria during cytokinesis, we surmised that Miro/Cenp-F interaction would have a special role during this cell cycle phase. We imaged late cytokinetic wild-type, as well as Miro-less and Cenp-F-silenced KERMIT cells. In wild-type cells (Fig. 3b, left) or cells lacking only Miro1 (Miro1^CRISPR^), the mitochondrial network adopted a stereotypical shape, where mitochondria were parallel and extended outwards and away from the division plane (arrowheads). In Miro-less cells, however, mitochondria remained clumped around the nucleus (shown by the ER marker in blue, Fig. 3b, right), and this was, again, phenocopied by Cenp-F silencing. Thus, Miro and Cenp-F are necessary for proper mitochondrial spreading after mitosis. Intriguingly, the mitochondrial spreading defect was accompanied by a defect in cellular spreading, which we measured as the minimal distance between the most distant ER signal and the nuclear envelope (Supplementary Fig. 4B). It was thus possible that the mitochondrial spreading defect observed both in interphase and cytokinesis in Cenp-F-silenced and Miro-less cells was caused by an altered cell shape. To ensure that the mitochondrial spreading defect was not due to a change in cell shape, we used Cytoo crossbow-shaped micropatterns to force cells into identical shapes. This allowed us to generate mitochondrial probabilistic density maps^18^. In cells lacking Miro or Cenp-F, mitochondria were more concentrated around the cell centre and failed to spread to the periphery (Fig. 3c). To assess the statistical significance of this phenotype, we computed the moment of inertia of the mitochondrial network around its centre of mass for every cell^19^. This metric, which reflects the spreading of the mitochondrial network, was reduced in cells lacking Miro or Cenp-F (Fig. 3d), confirming that Miro and Cenp-F are necessary for proper mitochondrial spreading, independent of cell spreading. To address whether the spreading defect was specific for a cell cycle phase, we co-stained cells with an anti-cyclin-A antibody. Interestingly, the spreading defect was visible in G1 as well as S/G2 cells (Supplementary Fig. 4A). This is surprising since Cenp-F is absent from G1, and indicates that G1 cells inherit a spreading defect from previous cell divisions.

Because of their reduced adherence, however, mitotic cells could not be forced into predetermined shapes by micropatterns. Therefore, whether the cytokinetic mitochondrial spreading defect is secondary to a change in cell shape remains unanswered. However, because of the results in interphase cells and since Miro proteins are mitochondrial, we consider more likely that defective mitochondrial spreading may, in fact, cause altered cell shape.

### Mitochondria distribution is linked to microtubule dynamics

**Figure 4:**
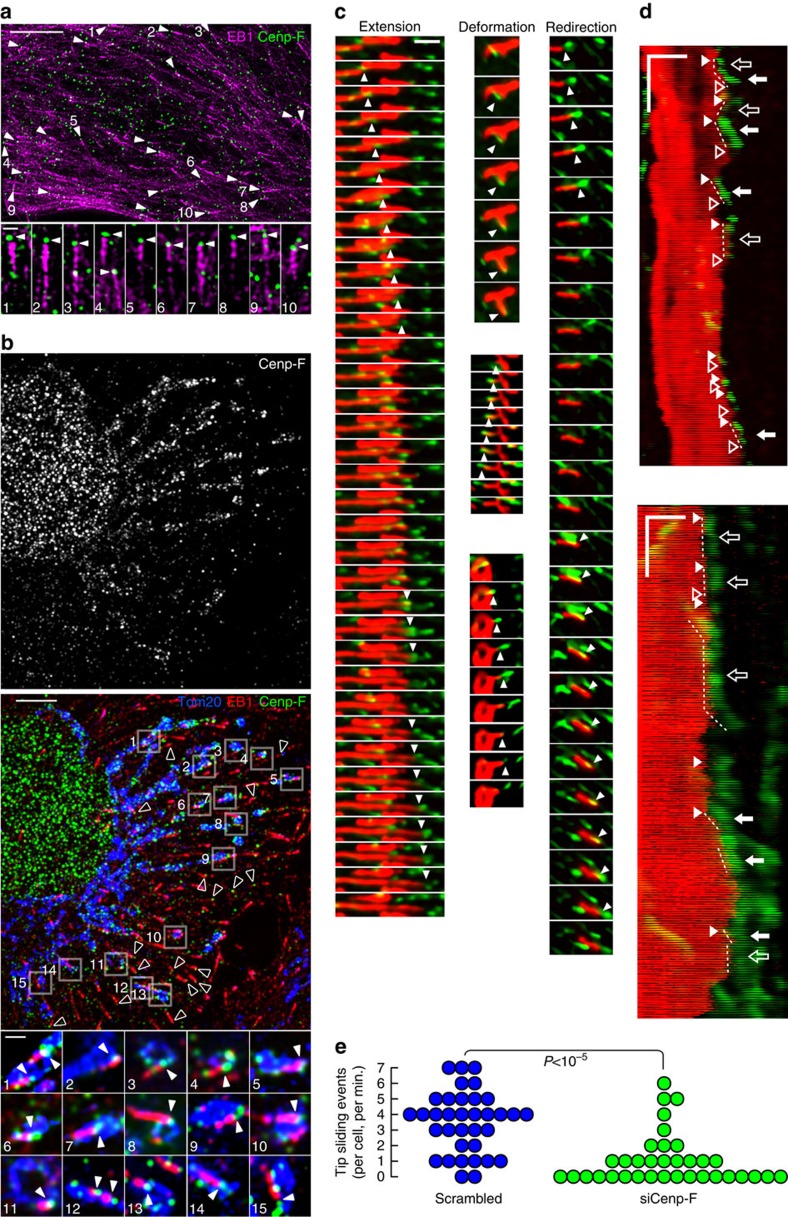
Mitochondria are connected to microtubule tips. (**a**) Structured-illumination microscopy of Cenp-F and EB1 in U2OS cells. Top: arrowheads represent example EB1 comets that have clear Cenp-F signal at their tips. Scale bar, 5 μm. Bottom: magnification of some of these comets. Scale bar, 500 nm. (**b**) As in **a**. Mitochondria were additionally revealed using an α-Tom20 antibody. Boxed areas in the top panel are magnified in the lower panels. Arrowheads represent Cenp-F signal at the tip of comets and on the mitochondria. Open arrowheads in top panel indicate EB1 comets that have Cenp-F signal at their tips, but are not on mitochondria. Note that the upper panel is a Z-projection across 2 μm, while the lower panels are individual sections. Scale bar, 5 μm (top), 500 nm (bottom). (**c**) Time-lapse images G2-synchronized of U2OS cells expressing GFP-EB1 (green) and mt-dsRED (red). Arrowheads show tip-tracking events. Timescale is 800 ms per frame. Scale bar, 2 μm. (**d**) Kymographs of time-lapse experiments as in **c**, showing that additionally to mitochondria tip-tracking events (plain arrows), microtubule rescue (plain arrowheads), pauses (open arrows) and catastrophes (open arrowheads) events are enriched at mitochondria tips. Scale bar, *x*: 2 μm, *y*: 10 s (0.4 s per frame). (**e**) Quantification of mitochondria tip-tracking events in cells treated or not with siRNA against Cenp-F. *P* value is from a Mann–Whitney–Wilcoxon *U*-test. The experiment has been repeated three times.

Both phenotypes observed in interphase and in cytokinesis indicated a defect in anterograde transport of mitochondria. This is paradoxical because Cenp-F is known to interact with Dynein retrograde motor complexes^11^. Dynein needs to be sent to the periphery before transporting cargo towards the cell centre. This involves association with growing microtubule tips^20^, a phenomenon known as tip-tracking^21^. Cenp-F binds to the microtubule tips at the kinetochores^22^, and associates with the microtubule end-binding protein 1 (EB1)^23^. We wondered whether Miro/Cenp-F could mediate anterograde mitochondrial movement through tip association. We imaged EB1 and Cenp-F using 3D-SIM. We focused on S/G2 cells, since they showed both appropriate Cenp-F signals and a flat morphology conducive to optical imaging. EB1 had a characteristic comet-like staining that marks the microtubule growing tips (Fig. 4a)^20^. In S/G2 cells showing a typical nuclear Cenp-F staining, Cenp-F was also often found in puncta at the extreme tips of EB1 comets (Fig. 4a). We found that 40% of all EB1 comets had visible Cenp-F staining at their tips. This proportion rose to 80% in cytokinetic cells (Supplementary Fig. 4C). To assess the significance of this localization, we computed the proportion of Cenp-F foci located in the vicinity of EB1 comet tips (less than three pixels, that is, the microscope resolution limit), and found that this proportion was much higher than that computed for random points on the microtubules (*P*<10^−7^ using a permutation test).

Cenp-F localization ahead of EB1 comets is remarkable and contrary to expectations for a hypothetical EB1-binding protein. This localization pattern is, however, not unprecedented: the CytosKeleton Associated Protein 5 (CKAP5/Ch-TOG) was also shown to lie at the tip of EB1 comets^24^. We compared the localization of Cenp-F and CKAP5/Ch-TOG, relative to EB1 and microtubules, and found both proteins to localize very similarly (Supplementary Fig. 4D–I).

We imaged mitochondria using an α-Tom20 antibody, together with EB1 and Cenp-F, and found that the comets whose tips were on mitochondria showed Cenp-F staining (Fig. 4b). These results were also true in cytokinetic cells (Supplementary Fig. 4C).

To test whether mitochondria could be moving together with growing microtubule tips, we imaged live G2-synchronized U2OS cells, which expressed GFP-EB1 and a mitochondrial marker (mt-dsRed). We could indeed observe numerous events where mitochondria moved along with growing microtubules. Such events were associated with extension, deformation and reorientation of individual mitochondria (Fig. 4c arrowheads; Supplementary Movie 1). Cenp-F was necessary for these events since their frequency was markedly reduced upon Cenp-F knockdown (Fig. 4e). Interestingly, we also observed that mitochondrial tips were local hotspots for microtubule dynamics events. Figure 4d and Supplementary Fig. 5 show that, in addition to coordinated microtubule tips and mitochondrial movements (plain arrows), rescue events (plain arrowheads), pauses (open arrows) and catastrophes (open arrowhead) happen preferentially at mitochondrial tips.

## Discussion

Thus, Miro-dependent Cenp-F recruitment governs intracellular mitochondrial distribution. We propose that Miro–Cenp-F interaction connects mitochondria to the tips of growing microtubules, causing mitochondria to track microtubule tips. The observation of tip-tracking mitochondrial movement is unprecedented, but is reminiscent of ER tip-tracking movements^25^. Both types of movement suggest that association with polymerizing microtubules can generate mechanical forces for membrane extension, deformation and reorientation^26^. It is unclear how such forces are generated, since plus-tip-bound proteins are usually turned over upon microtubule growth, and not dragged by the growing tip. A possibility is that motors, such as kinesins are involved in tip tracking. For instance, Cenp-E is a known tip-tracking kinesin^27^. It is worth noting that the mitochondria tip-tracking events that we observe are shorter than published ER tip-tracking events. This may be due to the fact that mitochondria are larger than ER tubules (∼500 versus ∼50 nm diameter) and therefore, more difficult to transport.

In turn, we observe that mitochondria influence local microtubule dynamics by promoting rescues, pauses and catastrophes. It is unclear how mitochondria can promote these events. One model is that the dragging forces imposed on growing microtubules by attached mitochondria may lead to pauses and catastrophes. Conversely, high Cenp-F local concentration at mitochondria tips may locally promote microtubule rescue.

It is intriguing that Cenp-F, a protein involved in chromosome segregation during mitosis, functions at mitochondria. Adaptors expressed during mitosis to move chromosomes may be reused for transporting other cargoes, such as organelles, at different cell cycle phases. Adaptor reuse for different structures, at different moments may emerge as a common theme. Indeed, we found that Cenp-F was often associated with EB1 comets even when it was not associated with mitochondria (Fig. 4b, open arrowheads; Supplementary Fig. 4C), suggesting that Cenp-F may also be involved in the anterograde transport of other cargoes. Intracellular signals may temporally and spatially direct the motors and adaptors to the appropriate cargoes. Such signals may be integrated either on Miro first GTPase domain, or directly on Cenp-F Miro-binding peptide, which harbours serine and threonine residues that are phosphorylated during the cell cycle^28^,^29^,^30^. These signals may promote or inhibit Miro binding in a timely fashion.

This unsuspected connection between mitochondria, Cenp-F and microtubule will need to be clarified by future investigations. In particular, understanding how Cenp-F is connected to microtubule tips will be highly interesting to understand not only mitochondrial inheritance but also other Cenp-F-dependent processes, such as kinetochore assembly and nuclear envelope breakdown.

Although our observations were made in symmetrically dividing cancer cells, the mechanisms described herein might be of particular importance during development, especially in asymmetrically dividing stem cells. Cenp-F mutations cause congenital malformation and microcephaly in humans^31^. The aetiology of these diseases is unclear and may pertain, at least in part, to Cenp-F mitochondrial function.

## Methods

### Plasmids

pAc-Sec61α-GFP^32^ was a kind gift from Gia Voeltz (University of Boulder, USA). GFP-Cenp-F plasmid^17^ was provided by Stephen Taylor (University of Manchester, UK). pcDNA3.1-mtBFP was generated by inserting mtBFP sequence digested with HindIII Xba1 into pcDNA3.1+plasmid. pcDNA5/FRT/TO-3XFlag-6his-Miro1 and pcDNA5/FRT/TO-3XFlag-6his-Miro2 were cloned by PCR amplification of Miro1 and Miro2 CDSs using primers #1 and #3 (see Supplementary Table 1), and 2# and #4, respectively, using complementary DNA clones as templates (IMAGE clones 40118340 and 4859240, respectively), followed by reamplification using primer #5, #3 and #5, #4, respectively. The PCR products were cloned using Gateway into the pcDNA5/FRT/TO plasmid (Invitrogen). GTPase and EF-hand mutants of Miro1 were generated by site-directed mutagenesis of the pcDNA5/FRT/TO-3XFlag-6his-Miro1 plasmid using primers #10 and #11 for the T18N mutation, #12 and #13 for the E208K mutation, #14 and #15 for the E328K mutation and #16 and #17 for the S432N mutation. Constructs containing different Cenp-F fragments (residues 1–979, 843–1764, 1756–3114, 1756–2375, 2375–3114, 2519–2831, 2719–2831 and 2819–3017) were prepared by PCR using the indicated primers (#22–#34, respectively), and using the GFP-Cenp-F plasmid as a template, followed by cloning into the pcDNA5/frt/to plasmid vector within BamHI and NotI restriction sites. Miro1 CRISPR plasmids (pX330-Miro1ex7 and pX330-Miro1ex8) were generated by cloning annealed oligos (#6, #7 and #8, #9, respectively), designed by the CRISPR design tool into pX330, as described^15^.

### Cell culture and transfection

HEK293 and U2OS cells were grown at 37 °C in DMEM (Life Technologies) supplemented with 10% FCS, 100 μg ml^−1^ streptomycin, 100 U ml^−1^ penicillin and 1% L-glutamine. To establish the KERMIT cell line, U2OS cells were cotransfected with linearized mtBFP and pAc-Sec61α-GFP. Individual clones were selected in 0.4 μg ml^−1^ G418 and maintenance was performed in 0.2 μg ml^−1^.

The 3 × flag-6 × HIS-Miro1/2 cell lines were created by cotransfection of pcDNA5/FRT/TO vectors encoding 3 × flag-6 × HIS-Miro1/2 with pOG44 into T-Rex-293 cells (Life Technologies), according to the manufacturer’s protocol. The clones were selected in 100 μg ml^−1^ Hygromycin (Invitrogen). For stimulating transgene expression, cells were treated with 10 nM doxycycline for 24−48 h.

GFP-EB1-expressing cells were generated by cotransfection of pcDNA5/FRT/TO-EGFP-EB1 (kind gift from Jochen Beck, ETH Zurich) into T-Rex-U2OS cells (Life Technologies), according to the manufacturer’s protocol.

### Flag-miro purification and mass spectrometry

T-Rex-293 expressing 3 × Flag-6 × HIS-Miro1/2 were harvested and homogenized in lysis buffer (20 mM Hepes, 150 mM potassium acetate, 2 mM magnesium acetate, 0.1 mM phenylmethyl sulphonyl fluoride, 1 μg ml^−1^ leupeptin, 1 μg ml^−1^ aprotinin and 1 μg ml^−1^ pepsatin), containing 2% Triton-100 × . Whole-cell extracts were incubated with 4% M2-Flag-coated magnetic beads (Sigma). The beads were thereafter washed six times with ice-cold lysis buffer, containing 0.2% digitonin and eluted with the same buffer containing 150 ng ml^−1^ 3 × Flag peptide. Samples were then diluted fivefold with 8 M urea and 0.1 M ammonium bicarbonate and reduced using 12 mM dithiothreitol, at 32 °C for 30 min followed by alkylation with 40 mM iodoacetamide at room temperature for 45 min. The samples were diluted fivefold with 0.1 M ammonium bicarbonate and proteins were digested to peptides by adding 1 μg of trypsin (Promega) and incubating overnight at 32 °C. A column packed with C-18 material (The Nest Group) was used to purify and concentrate peptides. The peptide samples were analysed on a 5600TripleTOF mass spectrometer (ABSciex) equipped with a nanoelectrospray ion source. Chromatographic separation of peptides was performed using an Eksigent Ultra nano LC system (ABSciex) coupled to a 15-cm-fused silica emitter, 75 μm diameter, packed with a Magic C18 AQ 5 μm resin (Michrom BioResources). Peptides were loaded on the column from a cooled (4 °C) Eksigent autosampler and separated with a linear gradient of acetonitrile/water, containing 0.1% formic acid, at a flow rate of 300 nl min^−1^. A gradient from 5 to 35% acetonitrile over 120 min was used. The mass spectrometer was operated in data-dependent acquisition mode. For TOF analyses, the accumulation time was set to 0.299995, s, and the mass range to 400–1250 Da. Peptides with 2–5 charges and signals exceeding 150 cps were selected for fragmentation. Per cycle, up to 20 precursor ions were monitored and excluded for 20 s after one occurrence. The product ion analysis was performed with an accumulation time of 0.149998, s, and a mass range of 170–1,500 Da in a high-sensitivity mode. The total cycle time was 3.35 s. Raw data were converted from WIFF format to MGF format (Matrix Science) using the AB SCIEX MS data converter (version 1.1 beta). Peak lists in .mgf format were searched against a human protein database downloaded from Uniprot ( http://www.uniprot.org/, September 2009) with Sorcerer-SEQUEST (Thermo Electron). Trypsin was set as the digesting protease with the tolerance of two missed cleavages, one non-tryptic terminus and not allowing for cleavages of KP and RP peptide bonds. The monoisotopic peptide and fragment mass tolerances were set to 50 p.p.m. and 0.8 Da, respectively. Carbamidomethylation of cysteins (+57.0214 Da) as defined as a fixed modification and the oxidation of methionines (+15.99492) as a variable modification. Protein identifications were statistically analysed with ProteinProphet (v3.0) and filtered to a cutoff of 0.9 ProteinProphet probability, which corresponded to a false discovery rate of <1%, calculated based on a target-decoy approach.

For SILAC labelling, Hek293 cells were grown for six generations in DMEM without arginine and lysine, and supplemented with either Arg^0^/lys^0^ for 3 × Flag6HMiro1 cells or Arg^10^ (^13^C_6_^15^N_4_)/Lys^8^ (^13^C_6_^15^N_2_) for the Hek293t-rex (control) cells. ‘Heavy’- and light’-labelled cells were harvested and directly combined in equal amounts before lysis. Flag purification, peptide preparation and analysis by mass spectrometry was preformed as above. For each peptide in which a light and heavy form was detected, the light to heavy ratio was determined by manually measuring the area under the curve using PeakViewer(version 2.3.3). Light and heavy peptides were identified and their ratios were calculated by MaxQuant^33^. The results are compiled in Supplementary Data Set 1. All uncropped original blots are shown in Supplementary Fig. 6.

### Yeast two-hybrid assay

The assay was performed according to the standard protocol^34^. Briefly, LexA-fused Cenp-F bait plasmid was created by gap-repair cloning of a PCR-amplified C-terminal fragment of Cenp-F (amino acids 2977–3020, primers #18 and #19, see Supplementary Table 1) into pEG202 vector linearized with NotI. The prey plasmid was created by gap-repair cloning of Miro1 (without transmembrane domain, amino acids 1–594, primers #20 and #21) or Miro2 (amino acids 1–592, primers #41 and #42) into pJG4-5 vector linearized with XhoI. Both plasmids were transformed into yeast strain EGY48 containing reporter plasmid pSH18-34, which encodes the lacZ reporter gene under control of LexA operators. Strain with empty bait and prey plasmids and strains with either Cenp-F bait or Miro1-prey plasmid alone were used as controls. Transformants were grown on -Ura/-Trp/-His media containing galactose to induce expression of prey protein. The transcription of the lacZ reporter was then assessed by the X-gal overlay assay^35^.

### Immunofluorescence and imaging

Cells were seeded on 18-mm coverslips in six-well vessels and optionally transfected with 6 μl lipofectamine-2000 (Invitrogen). On the next day, wells were optionally stained with 2 μM MitoTracker-Red-CM-H_2_Ros (Invitrogen) and washed in PBS (12 mM phosphate, 137 mM NaCl and 3 mM KCl, pH 7.4). For optimal mitochondria imaging, cells were fixed with 4% paraformaldehyde in PHEM buffer(60 mM pipes, pH 6.8, 25 mM Hepes, pH 7.4, 10 mM EGTA and 5 mM MgCl) for 10 min. Coverslips were washed three times in PBS and permeabilized in 0.5% NP-40, then washed three times with PBS and blocked in blocking solution (PBS+10% FCS+10 mg ml^−1^ BSA). For optimal cytoskeleton imaging, cells were fixed using 100% methanol (−20 °C for 8 min). Fixed cells were rinsed with PBS and incubated in blocking solution (5% FCS in PBS).

Primary antibodies were added to the blocking solution and incubated for 1 h. Antibody concentration: rabbit anti-Miro1 (HPA010687, Sigma) 1:500; mouse anti-Miro1 (WH0055288M1, Sigma) 1:500; mouse anti-Tom20 (clone 4F3, Abnova) 1:100; mouse anti-Phospho-H3 (ser 10, 9706, Cell Signaling) 1:1,000; mouse anti-Aurora-B (611082/3, BD Biosciences) 1:1,000; rabbit anti-Cenp-F (ab5, Abcam) 1:300; mouse anti-Cenp-F (610768, BD Biosciences) 1:500; mouse anti-alpha-Tubulin (B-5-1-2, Sigma) 1:1,000; rat anti-EB1 (KT51, Absea) 1:1,000.

Wide-field microscopy of fixed samples was conducted on a DeltaVision Microscope (IX-71; Olympus) connected to a camera (Roper CoolSnap HQ2, Photometrics), using a differential interference contrast Plan Apochromat × 60, numerical aperture (NA) 1.42 oil PlanApoN immersion objective. Three-dimensional (3D) image stacks were acquired in 0.2-μm steps using DAPI-FITC-TRC-CY5 filter set (Chroma). The 3D image stacks were deconvoluted with softWoRx (Applied Precision, LLC).

For Microtubule visualization, fixed cells were imaged using a DeltaVision OMX 3D-SIM Super-Resolution system controlled by DV-OMX software (Applied Precision). Images were captured at 0.125-μm step size with an UNIPLANAPO × 100/1.4 NA objective, using 1.514 immersion oil. The 405-nm-channel images were acquired for 100 ms at 1% laser strength, 488-nm-channel images for 50 ms at 10% strength, 561-nm-channel images for 50 ms at 100% strength and 642-nm-channel images for 50 ms at 100% strength. Images were processed using softWoRx (Applied Precision) and IMARIS 3D imaging software (Bitplane, Saint Paul, MN).

Colocalization analyses were performed using Daniel J White, Tom Kazimiers and Johannes Schindelin ImageJ plugin Coloc 2. Analysed regions of interest excluded the nucleus.

All experiments have been repeated a minimum of three times, with consistent results.

### RNAi

Oligonucleotides for siRNA synthesized against the target sequence of Cenp-F (oligo #1: 5′-CAGGAAAGACTAGCCCATATA-3′, oligo #2: 5′-CAGAATCTTAGTAGTCAAGTA-3′, oligo #3 (ineffective): 5′-CTGGTGATGGATTAACATATA-3′, oligo #4: 5′-ACCGAGAGAAATTGACTTCTA-3′), Miro2 (oligo #1 5′-AAGGCAGAGCTTTGGGCCAAA-3′, oligo #2 5′-GAGGTTGGGTTCCTGA-TTAAA-3′) and scrambled siRNA 5′-TTCTCCGAACGTGTCACGT-3′ were purchased from Qiagen. siRNA transfection were performed using Lipofectamine RNAiMAX (Life Technologies), according to the manufacturer, at a final concentration of 30 nM.

### CRISPR/Cas9 mutagenesis

To mutate endogenous Miro1, KERMIT cells were seeded on a 10-cm plate and cotransfected with 16.2 μg pX330-Miro1ex7, 16.2 μg pX330-Miro1ex8 and 3.6 μg HcRed plasmid (kind gift from Juan Gerez, ETH Zurich). At 48 h post transfection, Red fluorescent protein-positive cells were sorted by FACS and seeded upon a 10-cm plate at low density. Next, isolated colonies were transferred to a 96-well plate and genotyped.

### Live cell imaging

Inducible GFP-EB1-expressing cells were stimulated with 10 nM doxycycline for 16–24 h before imaging. G2 synchronization was performed by arresting cells with 2 μM thymidine 16 h before imaging. For visualization of the mitochondria, cells were transfected with mt-dsRed plasmid prior to doxycycline induction. Mitochondria and GFP-EB1 were imaged live at 37 °C using a DeltaVision OMX 3D-SIM Super-Resolution system controlled by DV-OMX software (Applied Precision), in epifluorescence mode. Images were captured with an UNIPLANAPO × 100/1.4 NA objective, using 1.514 immersion oil. Images were acquired every 400 ms for maximum of 2 min. All experiments have been repeated a minimum of three times, with consistent results.

### Mitochondrial spreading measurement

To analyse mitochondrial spreading we imaged and processed control and experimental cells identically, using ImageJ (Supplementary Note 1). Indicated cells were seeded onto a 22-mm^2^-micropatterned glass coverslip (Cytoo, Grenoble, France), according to the manufacturer’s protocol. At least 34 cells per condition were analysed. The centre of mass of the mitochondrial network was calculated using the following equation:




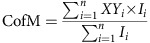




where *XY*_*i*_ represents the *XY* coordinate of each pixel and *I*_*i*_ represents the intensity of each pixel. Second, to determine the moment of inertia (M) of the mitochondrial network, the distance of each pixel to the centre of mass was computed:




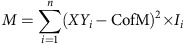




The measurement was performed by a MatLab function (Supplementary Note 2).

### Quantification of Cenp-F enrichment at microtubule tips

To assess whether Cenp-F localization at growing microtubule tips was due to chance, we devised the following analysis (Supplementary Note 3). Three channel images (EB1, Cenp-F and microtubules) were analysed. Only cytoplasmic areas were considered. First, using ImageJ, the position of the tips of EB1 comets (typically around 30 per image) were manually determined. The channel for Cenp-F was hidden during this manual step to avoid bias. We determined the position of Cenp-F foci automatically using the ‘Find Maxima...’ procedure (typically around 1,000 per images). We assessed the extent of comet tip and Cenp-F colocalization by counting the number of comets closer than 3 pixels (∼100 nm from a Cenp-F focus).

To assess whether the EB1–Cenp-F distances could be the result of a random draw, the distance of the closest Cenp-F focus was measured for random points of the microtubule network (typically 3,000 per image) picked using the ‘Find Maxima...’ procedure. Then random points (same number of points as the number of EB1 comets) were drawn 10^7^ times from this pool and the number of those colocalizing with Cenp-F was calculated. The *P* value equals the number of times the draw picked more random points colocalizing with Cenp-F foci than observed for comet tips, divided by the number of attempts.

### Quantification of microtubule tip-tracking events

Events where mitochondria move in a coordinate fashion with EB1 comets were counted manually after randomizing the ‘scrambled’ and ‘siCenp-F’ movies to avoid biases.

## Additional information

**How to cite this article:** Kanfer, G. *et al.* Mitotic redistribution of the mitochondrial network by Miro and Cenp-F. *Nat. Commun.* 6:8015 doi: 10.1038/ncomms9015 (2015).

## Supplementary Material

Supplementary Figures, Tables and Notes Supplementary Figures 1-6, Supplementary Table 1, Supplementary Notes 1-3

Supplementary Movie 1 Mitochondrial tip-tracking behaviour. Mitochondria (mt-dsRed, red) and EB1-GFP (green) were imaged together at 300 ms/frame. This movie is related to Fig. 4C.

Supplementary Dataset 1 Raw MS data from Miro1 and Miro2 purification
