# Supplementary material for: Mitotic redistribution of the mitochondrial network by Miro and Cenp-F
Source: Nat Commun. 2015 Aug 11;6:8015. doi: 10.1038/ncomms9015 (PMC4538849; doi:10.1038/ncomms9015)
Supplement: Supplementary Figures, Tables and Notes — Supplementary Figures 1-6, Supplementary Table 1, Supplementary Notes 1-3 [file ncomms9015-s1.pdf]

SUPPLEMENTARY FIGURES

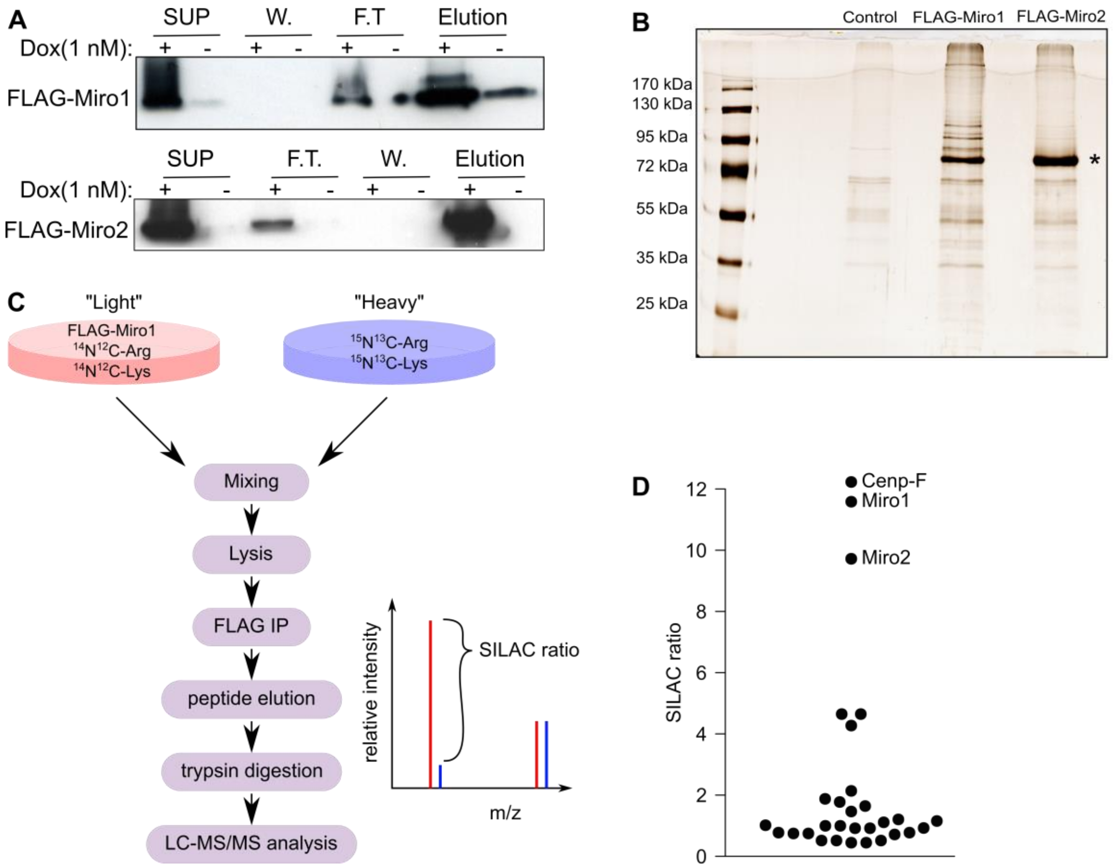

SUPPLEMENTARY FIG. 1

Purification of Miro interacting proteins.

(A) Western blotting with an α-Flag antibody of the following fractions from HEK293 cells expressing FLAG-Miro1 or FLAG-Miro2: whole cell lysate supernatant (SUP), flow-through unbound to the beads (F.T.), last wash (W) and elution. The expression of the transgene has been induced (+) or not (-) by the addition of 1 nM doxycycline (Dox).

(B) Silver-stained SDS-Page of the elutions from normal HEK293 (control), FLAG-Miro1 or FLAG-Miro2 expressing cells. The band indicated by an asterisk is the appropriate size for being FLAG-Miro.

(C) Stable isotope labeling with amino acid in culture (SILAC) strategy to discriminate proteins bound to Miro *in vivo* from those binding as a consequence of cell lysis. Cells expressing FLAG-Miro1 are cultured in a medium containing non-labeled Arginine and Lysine, while wild type cells are cultured in a medium containing ^{15}N-^{13}C (“heavy”)-labeled Arginine and Lysine. “Light”- and “heavy”-labeled cells are mixed before lysis, immunoprecipitation, and mass-spectrometry detection. The heavy label causes a mass shift of 8 Da for Lysine tryptic peptides and 10 Da for Arginine tryptic peptides. Proteins that were bound to Miro1 before lysis should be exclusively light-labeled, while proteins that bound to Miro1 after lysis should be equally light- and heavy-labeled. The SILAC ratio can thus be used as a proxy for specificity.

(D) Bee swarm plot of the SILAC ratios for proteins identified with high confidence in the immunoprecipitate.

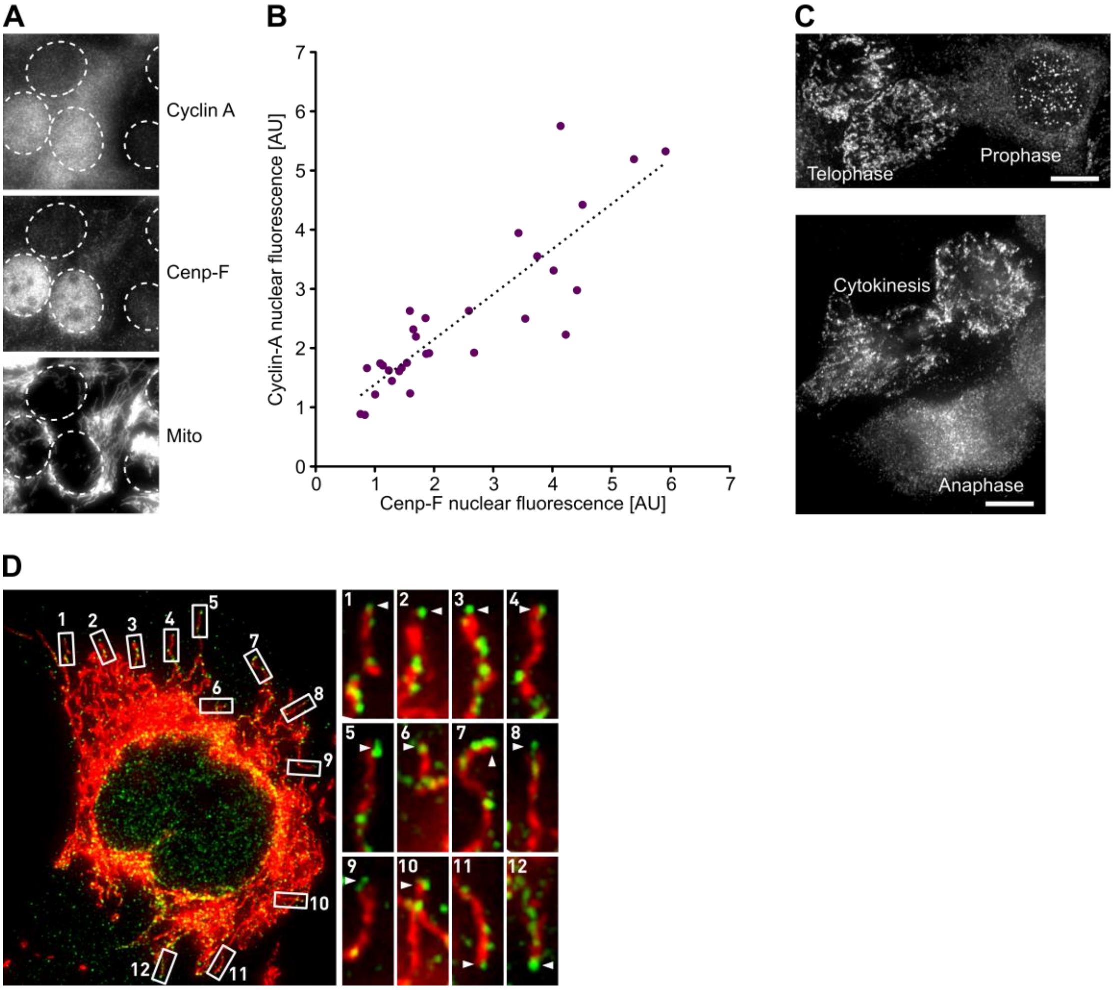

SUPPLEMENTARY FIG. 2

Cenp-F accumulates in the nucleus parallel to S/G2-specific protein Cyclin A

(A) Immunofluorescence of KERMIT cells with α-Cyclin A and α-Cenp-F antibody. Nuclei of the cells in the field of view are indicated by a dashed outline. The images represent average intensity projections over the whole thickness of the cells.

(B) Quantification of the fluorescence intensities of the nucleus in the Cenp-F channel (x-axis) and in the Cyclin A channel (y-axis). Each point represents one cell. AU: arbitrary units.

(C) Immunofluorescence of Cenp-F in cells undergoing mitosis. Two examples in which two mitotic cells were found in the same field of view show that the profound difference in Cenp-F recruitment between early and late mitosis is not simply due to variations in Cenp-F levels. Late mitosis cells (telophase and cytokinesis) show filamentous Cenp-F staining indicative of mitochondrial recruitment. By contrast early mitosis cells show diffuse staining (anaphase) or kinetochore and nuclear envelope staining (prophase). Scale bar, 10 µm.

(D) Late G2 cell imaged as in Fig. 1. Cenp-F staining is found at the nuclear envelope and the mitochondria, and is enriched at the distal tip of the mitochondria.

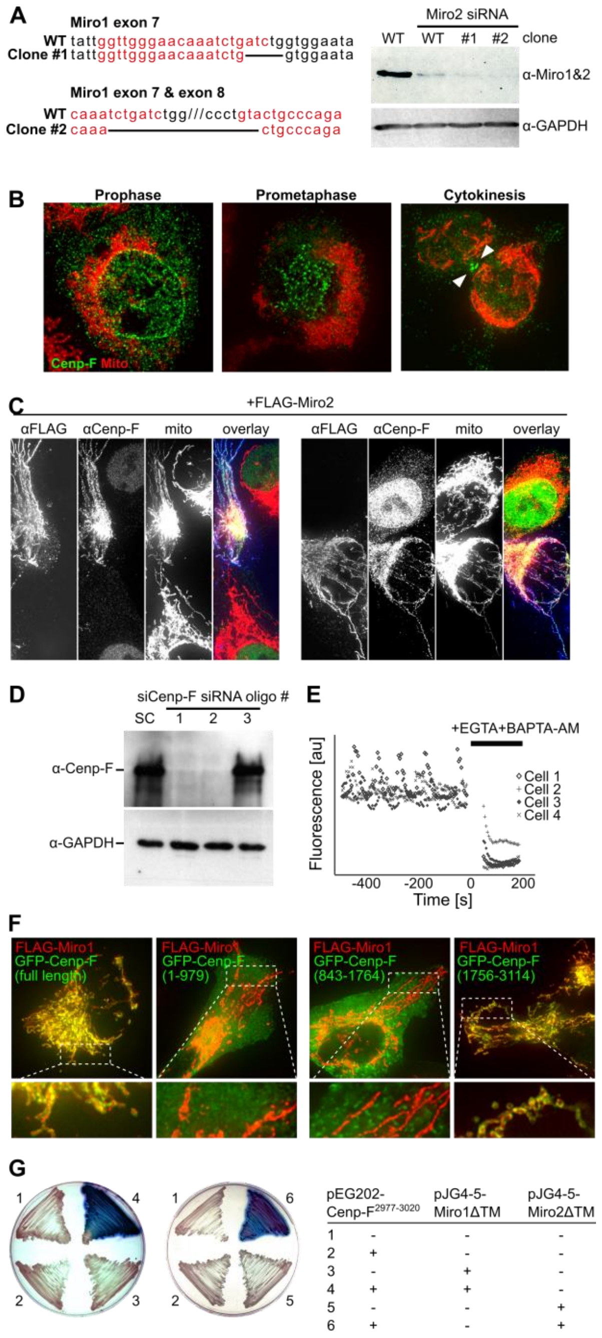

	pEG202-Cenp-F$^{2977-3020}$	pJG4-5-Miro1ΔTM	pJG4-5-Miro2ΔTM
1	-	-	-
2	+	-	-
3	-	+	-
4	+	+	-
5	-	-	+
6	+	-	+

SUPPLEMENTARY FIG. 3

Interaction of Miro and Cenp-F.

(A) Left: genetic lesions induced in clone #1 and #2 by CRISPR/Cas9 engineering. Red sequences are those used for generating the CRISPR guide RNA. Clone #1 bears a 5 bp deletion causing a frameshift in exon 7. Clone #2 bears a deletion spanning from exon 7 to exon 8, causing a deletion of 54 aminoacids and a frame-shift. Right, western blotting of whole cell protein extracts from normal KERMIT cells (WT), the same cells treated with siRNA against Miro2, or cells from Clone #1 and #2 treated with Miro2 siRNA. Top, detection with α-Miro antibody. Although this antibody is sold as an α-Miro1 antibody, it is raised against a peptide that is conserved between Miro1 and Miro2, and therefore recognises both. Most of the western signal actually comes from Miro2. Bottom: an α-GAPDH antibody is used as a loading control.

(B) Immunofluorescence of miro-less KERMIT cells showing unaffected Cenp-F localisation to the nuclear envelope in prophase (left), to kinetochores in prometaphase (middle) and to the midbody in cytokinesis (right).

(C) As in Fig. 2B, except that a FLAG-Miro2-encoding expression plasmid (instead of FLAG-Miro1) was transfected. FLAG-Miro2: blue, Cenp-F:green, mtBFP: red, in the overlay panel.

(D) Western blotting of whole cell protein extracts from KERMIT cells treated with scrambled siRNA (SC), or siRNA against Cenp-F (1, 2, 3). siRNA 3 obviously did not work and was not used further.

(E) EGTA +BAPTA-AM treatment effectively depletes cytosolic calcium and suppresses calcium oscillations. Cells loaded with fluorescent calcium indicator (Oregon Green Bapta-AM) were imaged in normal growth medium. EGTA and BAPTA-AM are added at timepoint 0. Measurements for four cells are shown. Cell 1 and 3 show spontaneous calcium oscillations.

(F) A FLAG-Miro1 expression plasmid was cotransfected with plasmids expressing GFP-fusion of full length or indicated fragments of Cenp-F. Cells were then fixed and stained with an anti-FLAG antibody. The full-length and the C-terminal fragment of Cenp-F are robustly recruited to mitochondria by FLAG-Miro1 overexpression. Bottom panels correspond to magnifications of the top panel (indicated by a white dashed box).

(G) Yeast-two hybrid assay by X-Gal overlay, using yeast strains bearing the indicated plasmids or their empty counterpart (pEG202 and pJG4-5, respectively).

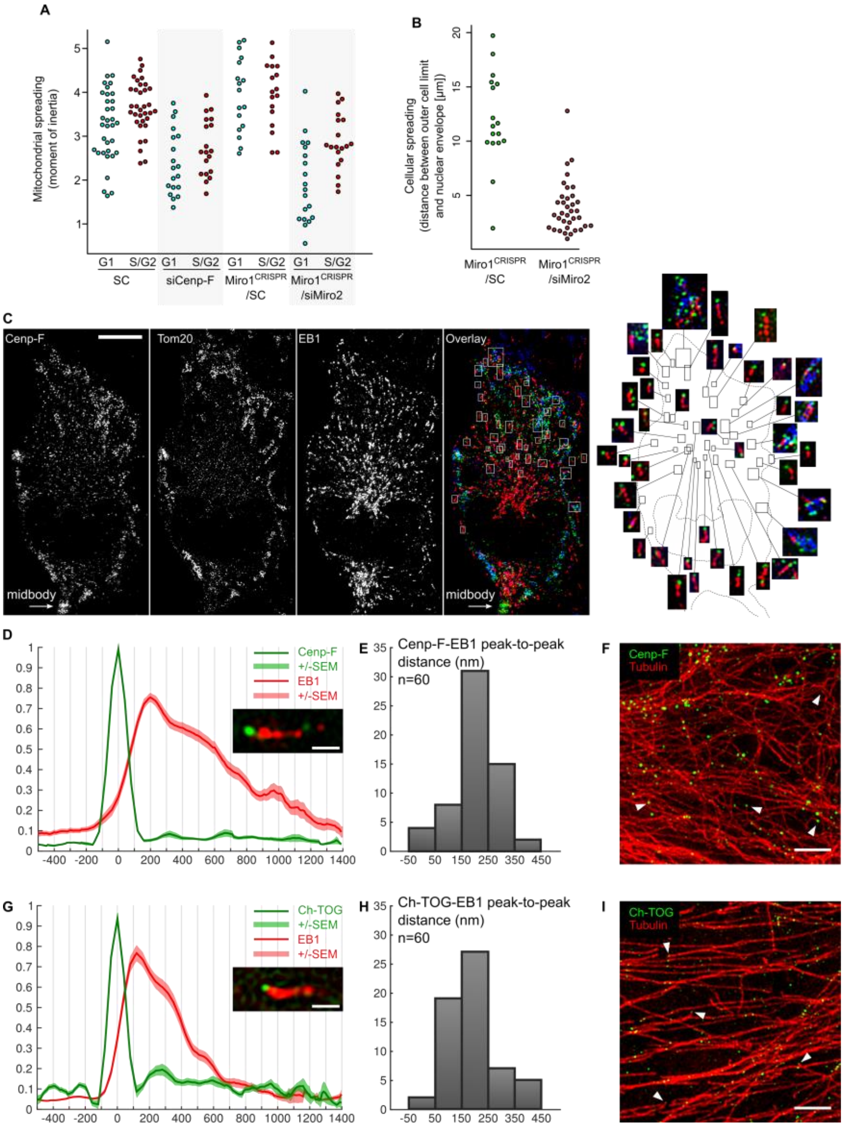

SUPPLEMENTARY FIG. 4

(A) Same data as in Fig. 3D except that cells were categorised as G1 or S/G2 according to the expression level of CyclinA.

(B) Miro-less cells have a cell spreading defect in cytokinesis. KERMIT cells were fixed, cytokinetic cells were imaged and the cellular spreading (defined as the minimal distance between the most distant point of the cell and the nuclear envelope) was measured in Miro1^{CRISPR} KERMIT cells (left) or Miro-less cells (right).

(C) Cytokinetic cell imaged as in Fig. 4B. Right, magnification of comets showing Cenp-F signal at their tips, which colocalises, or not with mitochondria. Scale bar, 5 μm.

(D, G) Comparison of Cenp-F and ch-TOG localisation. 3D-SIM immunofluorescence images of U2OS cells stained with Cenp-F and EB1 (D), or ch-TOG and EB1 (G) were acquired. The signal of each protein along the length of the comet was measured by line scanning. The position of maximal Cenp-F (respectively ch-TOG) signal was set at 0 nm. The intensities ± SEM are plotted. Scale bar, 500 nm.

(E, H) Peak-to-peak distance between the point of maximum Cenp-F (E) or ch-TOG (H) intensity and the point of maximal EB1 signal.

(F, I) 3D-SIM immunofluorescence images of U2OS cells stained with Cenp-F (F, green) or ch-TOG (I, green) and tubulin-α (red). Arrowheads indicate Cenp-F or ch-TOG signal at microtubule tips. Scale bar, 2 μm.

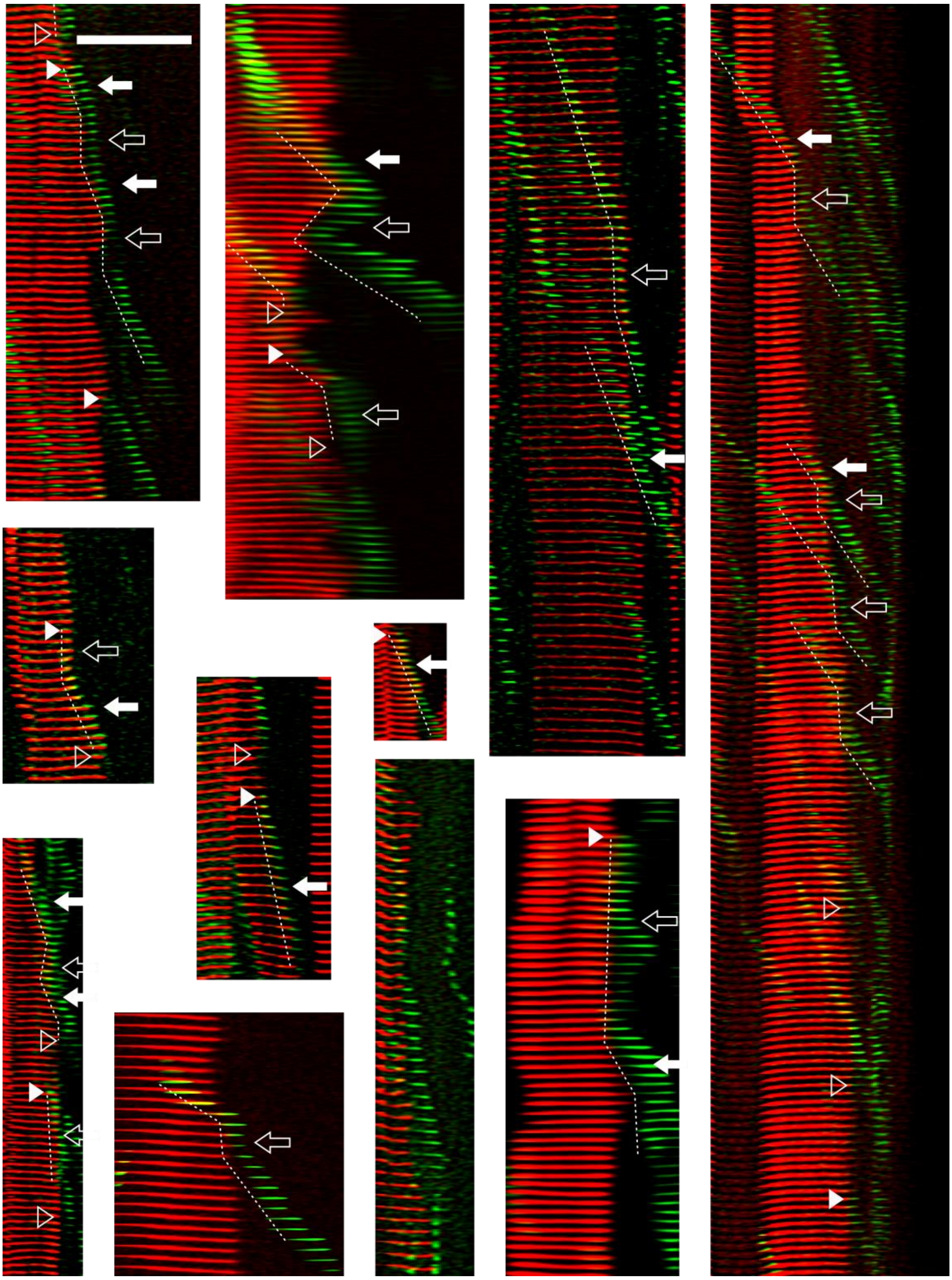

SUPPLEMENTARY FIG. 5

Representative kymographs of mitochondria (red) and EB1 (green) imaged as in Fig. 4C-D. Scale bar, 2 µm. Each step is 400 ms. Plain arrowheads: rescue event, open arrowheads: catastrophe, plain arrow: mitochondria-microtubule coordinated movements, open arrow: microtubule pausing at mitochondrial tips.

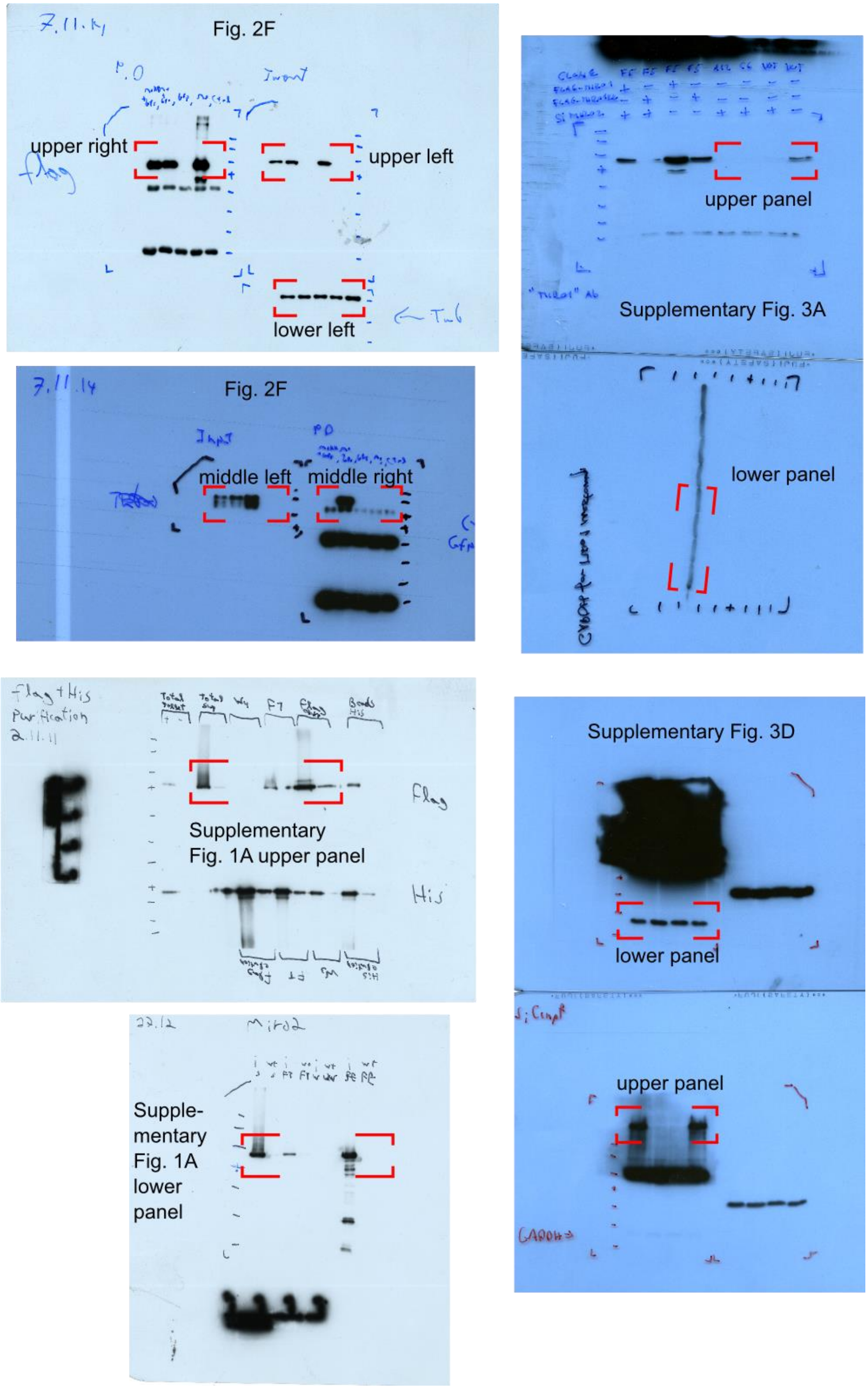

SUPPLEMENTARY FIG. 6

Non-cropped western blots for Fig. 2F and Supplementary Figs. 1A, 3A and 3D.

SUPPLEMENTARY TABLE 1:

Primers used in this study

#	Name	Sequence
1	3flag6hisMiro1	gactacaaggaccatgacggtgattacaaggatcatgacatcgactacaaggatgacgat gacaagcatcatcaccatcaccatggtatgaagaaagacgtgcg
2	3flag6hisMiro2	gactacaaggaccatgacggtgattacaaggatcatgacatcgactacaaggatgacgat gacaagcatcatcaccatcaccatggtatgaggcgggacgtgcgc
3	revMiro1Attb	ggggaccactttgtacaagaaagctgggtgtcatcgctgtttcaata
4	revMiro2attb	ggggaccactttgtacaagaaagctgggtgtcactggctcttcacca
5	Attbkozakflag	ggggacaagtttgtacaaaaaagcaggcttcaccatggactacaaggaccatgac
6	CRISPRmiro1ex7s	caccGGTTGGGAACAAATCTGATC
7	CRISPRmiro1ex7as	aaacGATCAGATTTGTTCCCAACC
8	CRISPRmiro1ex8s	caccCCTTCTCCTCTGGGCAGTAC
9	CRISPRmiro1ex8as	aaacGTACTGCCCAGAGGAGAAGG
10	hMiro1_T18N_F	cctagagttgggaagaactcactgattatgtc
11	hMiro1_T18N_R	gacataatcagtgagttcttcccaactctagg
12	hMiro1_E208K_F	ggtactctcaatgatgctaaactcaacttctttcag
13	hMiro1_E208K_R	ctgaaagaagttgagtttagcatcattgagagtacc
14	hMiro1_E328K_F	gctttgtcacctgataagcttaaagatttatttaaag
15	hMiro1_E328K_R	ctttaaataaatctttaagcttatcaggtgacaaagc
16	hMiro1_S432K_F	gaaaaactgtgggaaaaacggagttcttcaggc
17	hMiro1_S432K_R	gcctgaagaactccgtttttcccacagtttttc
18	CenpF2977F	GTTGGGGTTATTCGCAACGGCGACTGGCTGGAATTCgagccaga gggacttccagaa
19	CenpF3020R	TCGCCCGGAATTAGCTTGGCTGCAGGTCGACTCGAGtcataacttct gtgcagccaggcg
20	Y2H_pJG4-5_MIRO1_F	TACCCTTATGATGTGCCAGATTATGCCTCTCCCGAATTCGGCC GAatgaagaaagacgtgcggatcctgc
21	Y2H_pJG4-5_MIRO1_R	TGATTGGAGACTTGACCAAACCTCTGGCGAAGAAGTCCAAAGC TTtcaaagccaaaacgtggagctcttg
22	cenpf1_197bamh1F	gagaGGATCCATGAGCTGGGCTTTGG
23	cenpf1_197Not1R	tatGCGGCCGCtcaaagagtcccattctctt
24	cenpf_843-1764_fw	gagaGGATCCAAACAGATGAACTCAGACCTG
25	cenpf_843-1764_rv	taatGCGGCCGCtcaCCCCAGGAAATCCATAG
26	cenpf_1756_F	gagtGGATCCGGTCCTAATGCTTTGGTA
27	cenpf_3114_R	gtttGCGGCCGCtcactggaccttacagtt
28	cenpf_2375_fw	gagtGGATCCATGACCCAAAGTCTGAGA
29	cenpf_2519_fwd	gagtGGATCCAAGGATGAAGAAATCAGTAG
30	cenpf_2831_rev	tatGCGGCCGCtcaAGTACCTGTTTTCTGCTTCT
31	cenpf_2719_fw	gagaGGATCCCAGTATGAAGTAGAAATCCAG
32	cenpf_2831_rev	tatGCGGCCGCtcaAGTACCTGTTTTCTGCTTCT
33	cenpf_2819_fwd	gagtGGATCCCAAGCTGCACAGGAGAA
34	cenpf_3017_rev	tatGCGGCCGCtcaGGATAGCGCTAACTTCTG
35	CenpF2927F	GTTGGGGTTATTCGCAACGGCGACTGGCTGGAATTCaaagcttca ggcaagaggcaa
36	CenpF2957F	GTTGGGGTTATTCGCAACGGCGACTGGCTGGAATTCagcaagaa agcagtcatgagt
37	CenpF2977F	GTTGGGGTTATTCGCAACGGCGACTGGCTGGAATTCgagccaga gggacttccagaa
38	CenpF2987F	GTTGGGGTTATTCGCAACGGCGACTGGCTGGAATTCaaagggtttg ctgacatcccg
39	CenpF2987R	TCGCCCGGAATTAGCTTGGCTGCAGGTCGACTCGAGtcactttaca acttctggaagtcc
40	CenpF3003R	TCGCCCGGAATTAGCTTGGCTGCAGGTCGACTCGAGtcatcgcag gatatatgggctagt
41	Y2H_pJG_MIRO2_F	TACCCTTATGATGTGCCAGATTATGCCTCTCCCGAATTCGGCC GAatgaggcgggacgtgcgcatcctgt
42	Y2H_pJG_MIRO2_R	TGATTGGAGACTTGACCAAACCTCTGGCGAAGAAGTCCAAAGC

		TTtcaccggagccagaaggaagaggg
**43**	fw_Cenpf1_bhamh1	ccccctcgaggtcgacggtatcgataagcttgataGGATCCGCGATGAGCTGGGC
**44**	rev_Cenpf1	GCTGCAAAATACTTCATCTACACTGCAC
**45**	fw_cenpf2	AGTCTGCAGGAGGAGAATCTGACCAGGAAAGAAACC
**46**	rev_cenpf2_not	cggccgctctagaactagtggatcccccgggctgcgcggccgcTCACTGGACCTTACAGTTCT
**47**	Fw_GFP	CAAACCAGTGCAGTGTAGATGAAGTATTTTGCAGCatggtgagcaagggcgagga
**48**	Rev_GFP	GTTTCTTTCCTGGTCAGATTCTCCTCCTGCAGACTcttgtacagctcgtccatgc

SUPPLEMENTARY NOTES

SUPPLEMENTARY NOTE 1

ImageJ script to outline single cells and process the mitochondrial network for momentum of inertia analysis

```
name=getInfo("image.filename") rename("dd");
run("Split Channels");
//close non mitochondrial images run("Close");
run("Close");
//remove background, do a Z-projection (C1-dd is the mitochondria
channel, C2-dd is the ER channel )
selectWindow("C1-dd");
run("Subtract Background...", "rolling=12 stack");
run("Z Project...", "start=1 stop=40 projection=[Max Intensity]");
run("Subtract...", "value=100");
run("Enhance Contrast", "saturated=0.35");
selectWindow("C2-dd");
run("Z Project...", "start=1 stop=40 projection=[Max Intensity]");
run("Enhance Contrast", "saturated=0.35");
run("Merge Channels...", "c1=MAX_C1-dd c2=MAX_C2-dd create");
selectWindow("C2-dd");
close();
selectWindow("C1-dd");
close();
///// //setTool("polygon");
///loop repeated for as many cells as present on the picture.
ans=1;
i=0 while (ans>0){
      i=i+1;
      waitForUser("select the outline of a cell");
      run("Measure");
      run("Duplicate...", "title=Composite-1 duplicate channels=1");
      setBackgroundColor(0, 0, 0);
      run("Clear Outside");
      saveAs("Tiff", name+i+".tif");
      run("Close");
      Dialog.create("Test");
      Dialog.addMessage("do you have more cells to outline?")
      Dialog.addCheckbox("yes!", true) Dialog.show();
      ans=Dialog.getCheckbox();
      }
```

SUPPLEMENTARY NOTE 2

MatLab function to calculate the moment of inertia of the mitochondrial network.

```
function spread = Inertia(image)
imagesc(image)
%get the size of the image
[ysize xsize]=size(image)

%sum of all pixel values of the image
Isum=sum(sum(image));

%sum the image along the Y axis
for ii=1:ysize
        Ysum(ii)=sum(image(ii,:));
end

%center of mass (com) in Y
Ycom=0;
Ycom=double(Ycom);
for ii=1:ysize
        Ycom=Ycom+Ysum(ii)*double(ii);
end

%sum the image along the X axis
for ii=1:xsize
        Xsum(ii)=sum(image(:,ii));
end

%center of mass (CoM) in X
Xcom=0;
Xcom=double(Xcom);
for ii=1:xsize
        Xcom=Xcom+Xsum(ii)*double(ii);
end  CoM=[Ycom/Isum Xcom/Isum]

%spread=distance to CoM * pixel intensity, for each pixel of the
image.
spread=double(0);
for ii=1:ysize
      for jj=1:xsize
        spread=spread+sqrt((CoM(2)-double(jj))^2+(CoM(1)-
        double(ii))^2)*double(image(ii,jj));
      end
end
spread=spread/Isum
```

SUPPLEMENTARY NOTE 3

MatLab script to calculate the distance of eb1 comet to the closest Cenp-F focus. It uses three variables: eb1, list of XY coordinates for the tips of Eb1 comets; cenpf, list of XY coordinates of Cenp-F foci obtained by the “Analyse particles..” procedure in ImageJ; mt, list of XY coordinates for random points on microtubules (likewise acquired using the “Analyse particles..” in ImageJ.

```
clear disteb1 distmt
%for each eb1 comet, calculate the distance to the closest cenpf focus
for ii=1:length(eb1)
      disteb1(ii)=min(sqrt((cenpf(:,1)-eb1(ii,1)).^2+(cenpf(:,2)-
      eb1(ii,2)).^2));
end
%for each point on microtubules, calculate the distance to the closest cenpf focus
for ii=1:length(mt)
      distmt(ii)=min(sqrt((cenpf(:,1)-mt(ii,1)).^2+(cenpf(:,2)-
      mt(ii,2)).^2));
end
clear data
clear dist1p dist2p dist3p dist4p

%permutation test to find the probability that the eb1-cenp-f distances can be the result of a random draw.
iteration=10000000
for ii=1:iteration
%make a random draw of points on the microtubules
      for kk=1:length(disteb1)
        data(kk)=distmt(round(rand*(length(distmt)-1))+1);
      end
      %find the proportions of that are smaller than 1, 2, 3 or 4 pixels
      dist1p(ii)=length(find(data<1));
      dist2p(ii)=length(find(data<2));
      dist3p(ii)=length(find(data<3));
      dist4p(ii)=length(find(data<4));
end
clear onep twop threep fourp ii kk a b c d
%Compare the random draw to the data for eb1 comets.
a=length(find(disteb1<1))
b=length(find(disteb1<2))
c=length(find(disteb1<3))
d=length(find(disteb1<4))
length(find(dist1p>=a))/iteration
length(find(dist2p>=b))/iteration
length(find(dist3p>=c))/iteration
length(find(dist4p>=d))/iteration
```
